# Higher blood pressure and lower cardiac vagal activity in obese young individuals in supine and seated position

**Published:** 2017-09-23

**Authors:** André Rodrigues Lourenço Dias, Katrice Almeida de Souza, Laila Cândida de Jesus Lima de Sousa, Kamila Meireles dos Santos, Gabriel Kolesny Tricot, Jaqueline Alves de Araújo, Lucieli Teresa Cambri, Gisela Arsa

**Affiliations:** ^1^Graduate Program on Physical Education, Federal University of Mato Grosso, Cuiaba, Mato Grosso, Brazil; ^2^Graduate Program on Physical Education, Catholic University of Brasilia, Taguatinga, Federal District, Brazil

**Keywords:** Obesity, autonomic nervous system, heart rate variability, blood pressure, body position

## Abstract

**Background:**

Obesity triggers alterations in hemodynamic and autonomic control. There are few studies that investigate the effects of overweight and obesity in early adulthood on hemodynamic and autonomic variables.

**Aim:**

The aim of this study was to determine whether overweight and obesity in young individuals cause alterations in hemodynamic parameters and heart rate variability (HRV) in supine and seated position, and to correlate these variables with anthropometric features.

**Methods:**

Measurements were performed in 40 young untrained male study participants. The subjects were eutrophic (22.8 ± 0.3 kg/m^2^, N = 19), overweight (27.0 ± 0.5 kg/m^2^, N = 10), and obese (33.5 ± 0.8 kg/m^2^, N = 11). After 5 min in supine and seated position, the R-R intervals and blood pressure (BP) were recorded.

**Results:**

The systolic blood pressure were higher in overweight (supine, 122.9 ± 2.3 mmHg) and obese (supine, 123.9 ± 2.2; seated, 121.7 ± 2.3 mmHg) individuals compared to eutrophic individuals (supine, 111.8 ± 1.64; seated, 111.3 ± 1.8 mmHg) (p ⩽ 0.05). Obese subjects exhibited lower HRV (SD1, RMSSD, pNN50) compared to eutrophic individuals when seated. In obese subjects, the heart rate (HR) increased and HRV decreased (p ≤ 0.05) when seated versus supine position. The body mass, body mass index (BMI), and waist and abdominal circumferences correlated positively with BP (r = 0.40-0.64, *p* ≤ 0.05), while the BMI, waist circumference, BP, and HR were negatively correlated (r = -0.32 -0.62, *p* ≤ 0.05) with HRV (pNN50 and HF) in both body positions. BMI, waist circumference, BP and HR correlated negat- ively with additional HRV indices (SD1, SD2, RMSSD, TP, and LF) when seated.

**Conclusions:**

Obese and overweight individuals presented higher SBP, and obese individuals had lower HRV and cardiac vagal activity, associated with anthropometric variables.

**Relevance for patients:**

The monitoring of HRV in obese subjects in seated position allows improved prognosis of metabolic consequences to cardiac autonomic control.

## 1. Introduction

Each year, about 2.6 million people die from obesity-relatedproblems around the world. Global epidemiological statistics revealed that the prevalence of overweight and obesity in men was 36.9% in 2013 [1]. The obesity epidemic is associated with a plethora of health issues, which also affect children and adolescents [2]. Obesity contributes to the development of high blood pressure (BP), insulin resistance, and alterations in postprandial lipemia [3]. Neurophysiologically, obesity leads to reduced vagal nervous activity and maintenance or an in-crease in sympathetic nervous activity. Both affect heart rate (HR) and the baroreflex system, which detects and responds to changes in BP and corollary arterial dispensability and regu-lates HR accordingly [4].

The cardiac autonomic modulation (e.g., heart rate variabil-ity (HRV)) is partly controlled by body position. Relative to seated and standing positions, vagal nervous activity intensi-fies in supine position because the sympathetic nervous system signals peripheral vasoconstriction to ensure sufficient venous return to the heart [5]. This neurophysiological feedback mec-hanism is perturbed in obese children and adolescents, who exhibit decreased cardiac vagal activity and increased cardiac sympathetic activity in supine position [6, 7]. However, eu-trophic and obese children in standing and supine position do not show differences in HRV [8]. On the other hand, obese young adults exhibit a reduction in cardiac vagal activity and an increase in cardiac sympathetic activity in the supine posi-tion compared to their eutrophic counterparts [9]. Accordingly,the elevated cardiac sympathetic activity in a seated [10] or standing position [8] versus horizontal position may be exac-erbated in obese individuals, even when no increase in cardiac sympathetic activity is observed in the supine position in this population. In addition, different markers of metabolic syn-drome negatively influence HRV indices [11], as was observed in prepubescent children in whom visceral fat was related to decrease vagal nervous activity [7]. The body mass index (BMI) and BP were inversely associated with HRV in obese individuals [12], but this relationship was absent in overweight prepubescent children [7].

The number of studies that have analyzed overweight and obesity variables, metabolic syndrome markers, and cardiac autonomic modulation in children and young adults is limited.Existing research has mainly focused on treatment instead of integrative physiology on children and adolescents [7, 13], and on non-representative cohorts such as middle-aged adults diag-nosed with hypertension [14] and diabetes [15]. Presently it is unclear whether overweight and obese young adults with an otherwise healthy phenotype have reduced cardiac autonomic modulation in seated and supine positions. Obese sedentary individuals typically adopt the seated and supine position for a large part of the day. Neurologically driven cardiovascular issues as a result of the metabolic syndrome may hence have an early onset in these individuals [11]. Changes in cardiac autonomic modulation precede insulin resistance at the onset of metabolic syndrome [16] and increase the risk of the first cardiovascular event in individuals without cardiovascular disease by 32-45% [17].

The aim of this study was therefore to determine whether overweight and obesity in young individuals in supine and sea-ted positions is associated with perturbations in cardiac auton-omic modulation and hemodynamic parameters compared to eutrophic controls. The relationship between cardiac autono-mic, hemodynamic, and anthropometric parameters in both positions was studied by correlation analysis. The outcomes of this study assist in determining the effect of obesity-related metabolic complications on cardiac autonomic control.

## 2. Methods

### 2.1. Subjects and study design

The study was approved by the ethics committee on human research of the Julio Müller University Hospital under protocol number 391017/2013. Subjects who met the inclusion criteria and agreed to voluntarily participate in the study provided written informed consent prior to inclusion in the study.

The inclusion criteria were: (1) male sex, (2) willingness to provide information on family medical history, disease status,and use of medication during an anamnesis, (3) exhibiting physical activity reflective of otherwise good health (aside from being overweight or obese), and (4) the subjects were non-trained. A trained state was defined by engaging in regular exercise (performing a weekly routine containing at least two exercise sessions for a period longer than 30 days) for at least 4 months prior to the start of the study. The exclusion criteria were a BMI of < 18.5 kg/m^2^ or ≥ 40 kg/m^2^, tobacco use, and use of any medication that could interfere with the studied variables: anti-depressant drugs, alpha and beta adrenergic blockers, thermogenic agents, angiotensin receptor antagonists,ACE blockers, diuretics, and any other antihypertensive medi-cation.

The cohort consisted of 40 healthy, non-trained, male volu-nteers who were divided into 3 groups according to their BMI [18]: eutrophic (BMI 18.5-24.9 kg/m^2^; 20.8 ± 0.4 years; N =19), overweight (BMI 25.0-29.9 kg/m^2^; 21.9 ± 0.7 years; N =10) , and obese (BMI 30.0-39.9 kg/m^2^; 22.4 ± 0.4 years; N =11) .

**Figure 1.**
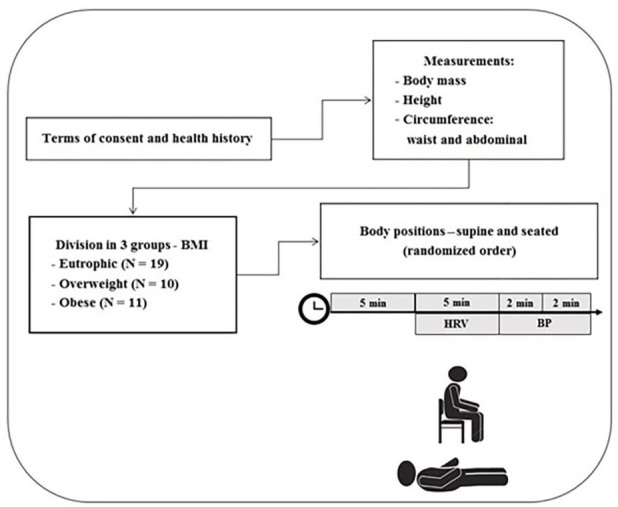
Summary of study design and procedures.

An overview of the study design is provided in Figure 1.

### 2.2. Procedures and morphometry

All procedures were performed at the NAFIMES Center for Physical Fitness, Computers, Metabolism, Sport, and Health of the Physical Education College of the Federal University of Mato Grosso. Subjects were instructed to eat 2 h before the beginning of procedures and to avoid physical exercise, stim-ulating foods and drinks such as alcohol and caffeine, and die-tary supplements that affect the cardiovascular system and hemodynamics during 24 h prior to their visit to the laboratory.Measurements were performed on single individuals.

Body mass (CAMRY EB9014, Säo José, Santa Catarina/Brazil) and height (SANNY stadiometer, ES2060, SBC, Säo Paulo/Brazil) were recorded for BMI determination. Waist (the smallest circumference of the thorax) and abdominal (the um-bilical level) circumference (CARDIOMED measurement tape)were measured to determine the cardiometabolic risk. Both circumferences predict the risk of developing of cardiovascular diseases, even after adjusting for BMI and several other car-diometabolic risk factors [19]. Next, participants were ran-domly asked to maintain a supine or a seated position in a si-lent and climatized room with a range of 22 and 25 °C for all experiments, while maintaining their usual breathing pattern.Seated subjects were instructed to keep the arms relaxed, the body leaning back, keeping hip and knees at a 90 degree angle,and the feet flat on the ground. Subjects in supine position were instructed to relax, keep the arms alongside the body,with elbows and knees extended. The subjects were requested not to speak, move, or sleep during the assessment period.Each body position was maintained for 10 min, 5 min to stabi-lize the HRV and 5 min to record the R-R intervals followed by two measurements of BP over a period of 4 min. The posi-tion was changed immediately after completing measurements in the first position.

### 2.3. Blood pressure and heart rate variability measurements

BP was measured twice on the left arm by the oscillometric method (model BP3T0-A, Microlife, Widnau/Switzerland)after 10 min in each body position, with an interval of 2 min between measurements. When the BP values differed by > 5%,a third measurement was performed, and the last two values were averaged. In the seated position the left arm was placed at the height of the heart in relaxed state.

The HRV index was obtained non-invasively using a HR monitor (model RS800cx, POLAR, Kempele/Finland) that Distributed under creative commons license 4.0 records the R-R intervals, reflecting the vagal and sympathetic components. This approach enables the assessment of the car-diac autonomic nervous system. The smaller the HRV, the lower the cardiac vagal activity and/or higher the cardiac sympathetic activity. The monitor records the R-R intervals using diverse analytical approaches for HRV determination,including analyses in the frequency domain and time domain [20] . The results acquired with a non-invasive HR monitor are in good agreement with results obtained by electrocardiogra-phy [21].

The HR monitor strap was placed on the chest over the lower third of the sternum and the HR receiver was placed near the subject. After a 5 min rest phase in the respective body position, the R-R intervals were recorded during 5 min as described in Task Force [22]. Artifacts and ectopics beats were removed from the R-R interval recordings using a moderate filter. HRV analysis was performed with Kubios HRV analysis software (Biosignal Analysis, University of Kuopio, Kuopio,Finland) using the autoregressive model in the time domain and frequency domain.

An exhaustive list of measured and calculated parameters is provided in Table 1.

### 2.4. Statistical analysis

Statistical analysis was performed in SPSS software version 20.0 (IBM, Armonk, NY/USA) and GraphPad Prism 6 version 6.01 (GraphPad Software, La Jolla, CA/USA). Descriptive data are present as mean ± standard error of the mean (SEM).

The quantitative data were tested for normality using the Shapiro-Wilk test and Levene's test for equal variance analysis.To assess differences between experimental groups was used One-way ANOVA with Bonferroni post-hoc test. To assess differences between experimental groups and body positions, a mixed repeated measures ANOVA with Bonferroni post-hoc test was employed. The Kruskal-Wallis test with Dunn's post-correction were used for the analysis of nonparametric data.Cohen's *d* was used as a measure of effect size for the main effects of inter-group differences (dgroup) and be-tween-position differences (dposition). Cohen [24] suggested that effect sizes of 0.2-0.49 are small, 0.5-0.79 are medium, and ⩾0.8 are large. Pearson's and Spearman's rank correlation analysis were employed to determine the relationship between parametric and nonparametric variables, respectively. Thresh-olds of 0.1, 0.3, 0.5, 0.7, and 0.9 for small, moderate, large,very large, and extremely large correlation coefficients were used [25]. A *p*-value of ⩽0.05 was considered statistically sig-nificant.

## 3. Results

### 3.1. Anthropometric parameters

The anthropometric data of the BMI-stratified groups are presented in Table 2. There were no intergroup differences with respect to age and height. In line with the body mass and BMI, the waist (WC) and abdominal (AC) circumferences were in the order of obese individuals > overweight individu-als > eutrophic individuals.

### 3.2. Hemodynamic variables

The hemodynamic and cardiac autonomic parameters ob-tained in the supine and seated positions in all groups are **Table 2. **Anthropometric parameters of subjects stratified by BMI.presented in Table 3. The HR and LF n.u. were higher in seat-ed position compared to supine position irrespective of BMI class (dposition= 2.04 and 1.32, respectively), whereas the SD1, pNN50, and HF n.u. were lower in the seated versus su-pine body position (dposition= 1.62, 1.74 and 1.57, respec-tively). In obese subjects only, SD2, RMSSD, TP, VLF were lower in seated subjects compared to subjects in supine posi-tion (dposition = 0.92, 1.06, 0.68 and 0.50, respectively).

With respect to intergroup differences, HR and SBP were higher in obese subjects in both positions compared to eu-trophic subjects, but did not differ between the overweight and obese group regardless of the position (dgroup= 0.96 and 1.70,respectively). The SBP was also higher in overweight supine subjects compared to matched controls. In seated obese sub-jects, the SD1, SD2, RMSSD, and pNN50 were lower relative to seated eutrophic subjects (dgroup= 0.85, 0.67, 0.86 and 1.06 respectively).

### 3.3. Correlation between anthropometric, hemodynamic, and cardiac autonomic variables in supine position

The correlation between hemodynamic, anthropometric,and autonomic parameters in the supine position is presented in Table 4. There was a moderate-to-large positive relationship between hemodynamic variables (HR, SBP, DBP) and anthro-pometric variables (body mass, BMI, waist circumference,abdominal circumference). Moreover, HR was negatively cor-related (moderate) with cardiac parasympathetic activity (SD1,RMSSD, pNN50). There was an inverse relationship between cardiac parasympathetic activity and anthropometric variables,as evidenced by the moderate negative correlation between the pNN50 and body mass, BMI, and waist circumference as well as by the moderate negative correlation between HF and BMI.

### 3.4. Correlation between anthropometric, hemodynamic, and cardiac autonomic variables in seated position

In the seated position the relationships between hemodynamic,anthropometric, and autonomic parameters were more pro-nounced compared to the supine position (Table 5). In line with extensive literature [9, 26, 27], all tested hemodynamic parameters exhibited a strong positive correlation with an-thropometric variables.

The relationship between hemodynamics and autonomic variables was mainly negative. For example, HR was inversely related (moderate to large) to multiple cardiac parasympathetic activity variables (SD1, RMSSD, pNN50, TP, HF, and HF n.u.)and HRV (SD2), with the exception of LF and sympathovagal balance (LF/HF ratio), where a positive relationship was found.BP chiefly followed the same trend, and was additionally neg-atively correlated (moderate) with VLF and LF (cardiac sym-pathetic and parasympathetic activity [22]).Lastly, all anthropometric parameters were inversely corre-lated (moderate to large) with SD1, SD2, RMSSD, pNN50, TP,LF, and HF, further underscoring the more significant impact of the seated position on physiology, and mainly the autonom-ic nervous system in terms of cardiac sympathetic and para-sympathetic activity.

**Table 1.**
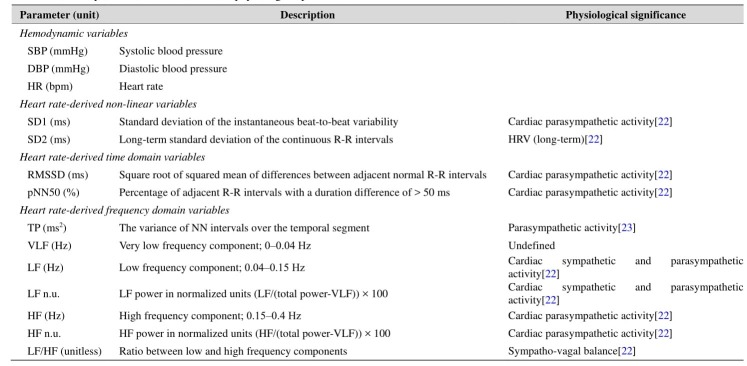
Summary of study design and procedures.

**Table 2.**
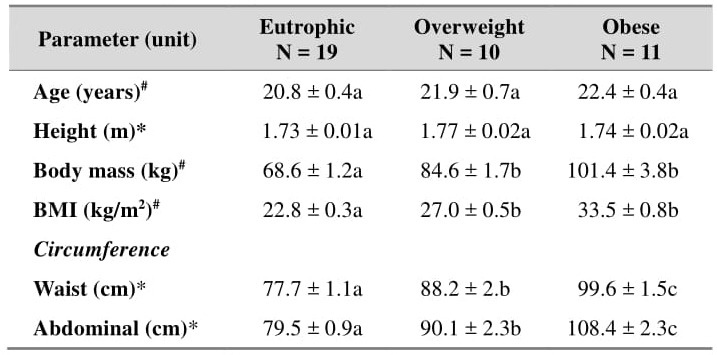
Anthropometric parameters of subjects stratified by BMI.

**Table 3.**
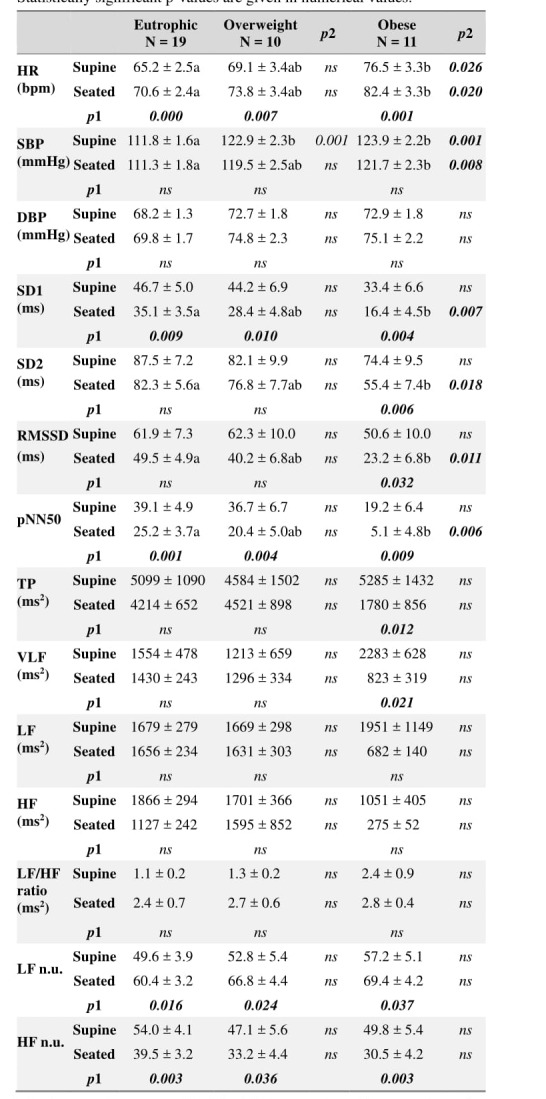
Hemodynamic and cardiac autonomic parameters per group. Statistically significant p-values are given in numerical values.

**Table 4.**
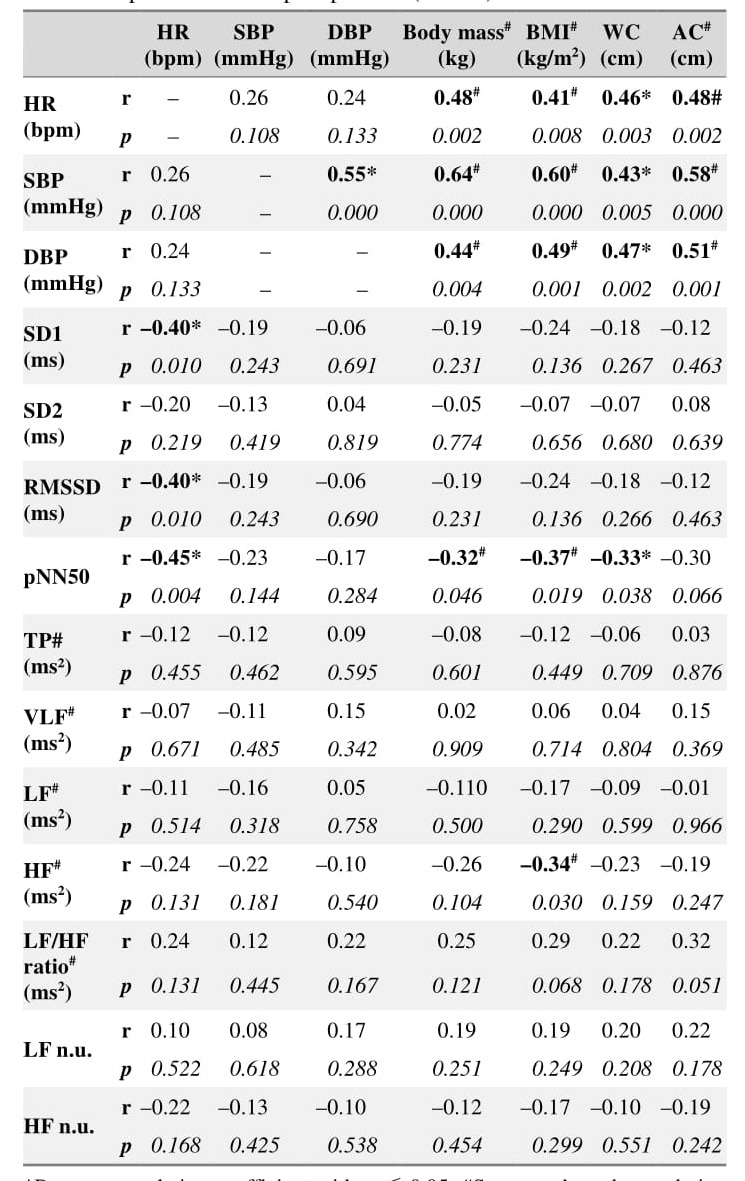
Correlation analysis between hemodynamic, anthropometric, and autonomic parameters in supine position (N = 40).

**Table 5.**
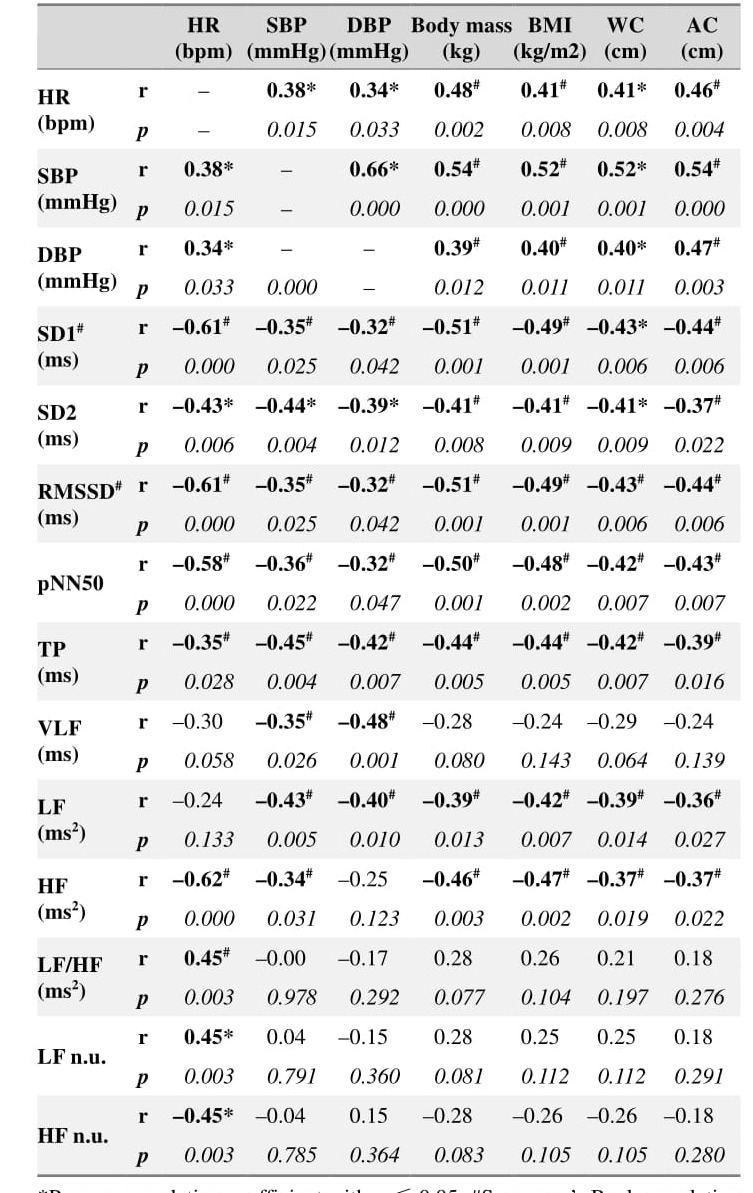
Correlation analysis between hemodynamic, anthropometric, and autonomic parameters in seated position (N = 40).

## 4. Discussion

The main results of this study were that (1) young obese in-dividuals have a higher HR and SBP regardless of body posi-tion compared to eutrophic controls; (2) young obese subjects exhibit lower vagal activity in the seated position compared to eutrophic individuals; (3) autonomic parameters are not as exacerbated in young overweight people as in their obese counterparts, although overweight subjects experience compa-rably elevated SBP in supine position as obese individuals compared to eutrophic controls; and (4) the anthropometric and hemodynamic parameters are negatively associated with cardiac vagal activity, especially when seated.Similar results have been reported for young obese people (~20 years old) in supine body position. These individuals exhibited higher SBP and HR and lower HRV and cardiac vagal activity when compared to their eutrophic counterparts [9, 28,29]. These responses have been mirrored in other age groups as well, such as obese children and teenagers, who displayed lower HRV and cardiac vagal activity in supine position [28-30]. Obese middle-aged adults had higher SBP in orthos-tatic position and lower cardiac vagal activity in supine posi-tion [31].

Studies suggest that the activity of cholinergic anti-infl-ammatory mechanism mediated by the vagus nerve could be evaluated by HRV [32, 33]. Corroboratively, the CARDIA study [34] found that HRV in seated position is inversely associated with a pro-inflammatory state in young adults. In obesity, the reduction in vagal activity and its cholinergic an-ti-inflammatory action may imply an increase in inflammation and metabolic complications [35]. In addition, the increase in SBP and DBP was associated with decreased vagal nerve ac-tivity in seated position in our study. Cardiac vagal activity has an important function in hemodynamic homeostasis, as evi-denced by compromised BP stability in the absence of cardiac vagal activity [36]. These findings may explain, in part, that cardiac vagal activity is negatively associated with insulin,glycemic and lipid profiles, SBP, DBP, and HR in different groups and pathologies [11, 15].

The lower cardiac vagal activity in young obese people precedes the increase in cardiac sympathetic activity. Auto-nomic cardiac dysfunction reduces left ventricular function,mechanic and electrical ventricular function, and coronary flux as is commonly observed in patients with heart failure [37, 38].The vagus nerve stimulation technique [39] results in partial reversal of these pathological symptoms in subjects with heart failure 12 months after stimulation therapy. Specifically, the symptom reversal is characterized by an increase in cardiac vagal activity and HR reduction [40], reduced sympathetic nervous system activity in healthy individuals [41]. These data show the importance of vagal activity in the treatment of car-diovascular diseases.

Although the young obese subjects had lower cardiac vagal activity, adjustments in autonomic cardiac modulation follow-ing the change in body position were observed between groups,corroborating results found in adults between 18-35 years who changed from prone to seated position [10]. This decrease of the HRV in the seated position occurs due to a lower venous return that results in the reduction of vagal nervous activity and increased sympathetic nervous activity, with a consequent increase in HR to maintain an adequate cardiac output and blood flow to the brain [10].

The change from supine body position to standing body po-sition leads to baroreflex adjustments that result in decreased vagal nervous activity and increased HR [42]. The magnitude of adjustments in autonomic modulation obtained in this study (SD1, pNN50, LF n.u., and HF n.u. indices in all groups), and probably in baroreflex control, can be lower with the changed of seated to supine position than the adjustments obtained to the changed of the standing to supine position, for example.

Our results appear to demonstrate loss of baroreflex sensi-tivity in overweight and obese young people with changing body position. However, SD1, SD2, RMSSD, and pNN50 were lower in seated position in obese individuals compared to eutrophic individuals. These findings may be related to decre-ased/lossed baroreflex control, reducing the cardiac vagal acti-vity [43], and worsen with increased carotid arterial stiffness [44,45]. Given that baroreceptors are mainly stretch receptors, ca-rotid arterial stiffening, a common condition in obese individ-uals [44], may reduce the stimulation of baroreceptors in re-sponse to changes in BP in consequence to the lower arterial compliance [45].

The body mass, BMI, waist and abdominal circumferences were negatively correlated with SD1, SD2, RMSSD, pNN50,TP, LF, and HF corroborating Lee et al. [46] revealing that vagal nerve activity negatively associated with intramuscular ectopic fat measured in thigh. Koenig et al. [47] observed a negatively correlation between cardiac vagal activity indices (pNN50 and RMSSD) with BMI, waist circumference, and waist-to-height ratio in 8,538 participants between 30 and 50 years old.

The associations between BMI and waist and abdominal circumference and cardiac autonomic indices occurred mainly in the seated position, suggesting that this position triggers the most profound changes in cardiovascular system function in obese individuals. Laederach-Hofmann et al. [12] analyzed HRV in seated position in middle-aged individuals classified from overweight to morbidly obese using frequency-domain indices, and observed that the higher BMI and waist circum-ference are related to lower LF index, and the higher waist-hip ratio to lower HF index.

Data from Kim et al. [48], Nemezio et al. [49], and Farah et al. [50] reinforce our data. The HRV indices in the supine po-sition are not the most sensitive measures of changes in cardi-ac autonomic modulation in overweight and obese individuals.Instead, the analyses seem to perform better in the seated posi-tion. The lower venous return in the seated position (due to the greater influence of gravity) may require more refined control of circulation compared to the supine position [51]. This translates to more profound changes in cardiac autonomic modulation in obese individuals.

However, the fat mass and fat mass percentage exhibited a negative correlation between some of the HRV indices in overweight and obese children in supine position [19]. Con-versely, BMI was negatively correlated with RMSSD and pNN50, but not with the indices in the frequency-domain [46].Likewise, body weight and waist circumference, but not BMI,were significantly correlated with indices of HRV and cardiac vagal activity in supine position [47, 52]. Therefore, the supine position to a lesser degree than the sitting position also shows an association of the anthropometric parameters with the cardiac autonomic modulation.

With respect to BP, high values in the overweight and obese group in supine position were found. Moreover, a significant relationship was found between body mass, BMI, and waist and abdominal circumference and BP at both positions. Simi-larly, overweight young adults with metabolic syndrome ex-hibited a higher BP relative to their eutrophic pairs, but the muscular sympathetic nerve activity was not different [53],evidencing the effect of excess body mass on blood pressure even in the absence of sympathetic over activity.

Kappus et al. [44] ascribed the higher BP in young obese people to higher carotid intima-media thickness and carotid arterial stiffness, while Lee et al. [46] reported the higher BP in overweight people may be related to high intramuscular ec-topic fat. The larger size and greater number of fat cells aug-ment the excretion of angiotensinogen [54] and consequently the production of angiotensin-I and angiotensin-II. These pep-tide hormones regulate physiological processes that result in increased BP, namely by promoting systemic vasoconstriction and the release of aldosterone by the gland, which affect kid-ney function by modulating renal perfusion and increasing sodium and water reabsorption [55].

Our study comes with limitations. Firstly, we did not moni-tor any variables when changing between the positions, which could yield additional information regarding accommodative changes in these autonomic indices. We chose to assess the maintenance period in these positions because sedentary indi-viduals remain in the supine or seated positions for long peri-ods. Nevertheless, we advocate follow-up studies that evaluate these positions for longer durations as well as autonomic ad-justments in other positions following postural changes. In addition, an investigation into BP variability may yield addi-tional information, given that the BP was higher in overweight and obese young people. Lastly, the small sample size is also considered a limitation of study.

### 4.1. Clinical implications

Measurement of HRV is non-invasive, easy, and can be performed at low cost. HRV has been investigated under dif-ferent conditions as rest, during exercise, and in healthy people and patients with cardiovascular disease [11, 22]. A lowered HRV and vagal activity are associated with an increase in morbidity and mortality, negatively affecting ventricular func-tion, coronary blood flow [29], and endothelial function [56].In our study, the seated position resulted in lower vagal activity,which was exacerbated in obese young people, and a higher BP.Accordingly, the seated position translates to a loss in cardiac autonomic control that may ultimately impair cardiac and en-dothelial function and augment the risk of morbidity in obese individuals. Consequently, HRV monitoring in the seated posi-tion enables us to better prognosticate metabolic complications of obesity in the context of cardiac autonomic control.

## 5. Conclusions

Obese but not overweight young people present some de-gree of cardiac autonomic impairment, but with the expected adjustments in cardiac autonomic modulation to supine and seated positions. However, overweight and obese young indi-viduals exhibited elevated BP compared to eutrophic young people. Additionally, BMI, waist and abdominal circumference were associated with BP in both positions. These variables along with HR were negatively associated with HRV indices mainly in the seated position. Accordingly, the seated position seems to be more prone to trigger changes in cardiovascular system functions. Given the impact of obesity on HRV indices and BP, changes in lifestyle (physical exercise and diet) are recommended so as to reduce the risk of obesity-related car-diovascular morbidity.
